# The use of Haemostatic Agents does not impact the rate of hemorrhagic complications in patients undergoing partial nephrectomy for renal masses

**DOI:** 10.1038/srep32376

**Published:** 2016-08-30

**Authors:** Yasmin Abu-Ghanem, Zohar Dotan, Issac Kaver, Dorit E. Zilberman, Jacob Ramon

**Affiliations:** 1 Dept. of Urology, Sheba Medical Center, Israel; 2 Sackler Faculty of Medicine, Tel Aviv University, Tel Aviv, Israe

## Abstract

Hemostatic agents(HAs) have gained increasing popularity as interventions to improve perioperative haemostasis and diminish the need for allogeneic red cell transfusion(PBT) despite a paucity of data supporting the practice. The aim of the current study is to examine the efficacy of HAs in reducing the rate of hemorrhagic complications during partial nephrectomy(PN). Data on 657 patients, who underwent elective PN between 2004–2013, were analyzed. The impact of HAs and SURGICEL was evaluated by comparing four sequential groups of patients: Group1 = Sutures alone, Group2 = sutures and HA, Group3 = sutures and SURGICEL, Group4 = both HA and SURGICEL. Complications included post-operative urinary leak(UL), PBT rate, delayed bleeding and post-operative renal failure. Results showed that the use of HAs did not engender a statistically significant difference in overall complications rate. Specifically, the addition of HAs did not reduce the rate of PBT, delayed bleeding or UL. Further analysis revealed that patients who received SURGICEL had significantly higher PBT rate and higher prevalence of UL cases. Addition of HAs to SURGICEL had no effect on the rate of these complications. In the current study, the use of HAs during open and laparoscopic PN did not reduce the rate of negative outcomes. Adequate suture renorrhaphy may be sufficient to prevent hemorrhagic complications.

In recent years, partial nephrectomy (PN), and specifically laparoscopic PN (LPN) has become the standard of care in the management of selected renal lesions^1^. Initially, LPN was proffered only in the case of a small, peripheral, exophytic tumor. However, with increasing experience, the application of LPN has extended to tumors invading more deeply into the renal parenchyma up to the collecting system or renal sinus^2^,^3^. Nevertheless, as laparoscopic surgeons approach more difficult tumors, the complexity of tumors requiring Open PN (OPN) is even further magnified. In both approaches, the excision of such deeply infiltrating tumors routinely necessitates division of major intrarenal vessels and precise entry into the collecting system to ensure an adequate margin of resection. Such resections are therefore associated with significant risks of bleeding and urinary leak^3^.

Despite the relatively low incidences of hemorrhagic complications requiring transfusion after PN, it remains one of the most serious complications^4^. With many reports suggesting that allogeneic blood transfusion is associated with an adverse outcome^5^,^6^,^7^, several attempts have been made to reduce the risk of bleeding. In the past decades, a wide variety of haemostatic agents (HAs) has been developed as surgical tools, in order to reduce the rate of such complications. Different tissue adhesives (also called glues) have been used to assist in haemostasis and collecting system closure during open and laparoscopic PN^2^,^8^,^9^,^10^,^11^,^12^. Yet, despite the growing clinical application of HA during PN, there is little data regarding their cost-effectiveness in preventing major complications^13^. In this study, we present an analysis of the effectiveness of using HAs and glues in laparoscopic and open PN.

## Patients and Methods

A total of 657 consecutive patients underwent PN at our institution between 2004 and 2013. All operations were performed in the same surgical environment (four surgeons operating in one medical center). Patient demographics and surgical details were collected retrospectively, following an approval given by the Sheba Medical Center Institutional Review Board (IRB)/Ethics (Helsinki) Committee, in accordance with relevant guidelines and regulations. The need for informed consent was waived by our IRB. All patients were considered for a comparative study, and four groups were defined on the basis of the method of haemostasis used during the procedure. Group 1 = renorrhaphy was completed using sutures alone in 147 patients (22.4%); Group 2 = both sutures and HA were used in 26 patients (3.9%); group 3 = sutures and SURGICEL (Ethicon, Somerville, NJ, USA), without HA in 183 (27.8%) patients; and group 4 = sutures, HA and SURGICEL in 301 (45.8%) patients.

Altogether, 327 patients received HA (with or without SURGICEL). Of those, 290 (88.6%) received cryoprecipitate, prepared from a single allogeneic donor, combined with commercially prepared thrombin. The remaining patients received commercial sealants (bovine serum albumin–based adhesive, BioGlue; Kennesaw, GA, USA). In all cases, HA were used by covering the renal injury immediately after performing sutured renorrhaphy. Patient demographics and operative details were collected retrospectively. Clinicopathologic variables recorded included: age, gender, height, weight and Body Mass Index (BMI), preoperative hemoglobin and creatinine levels, receipt of perioperative blood transfusion (PBT) and number of units transfused. Pathology related variables included: tumor location, multifocal mass, central or hilar tumor, mass size, as well as tumor pathology- malignant tumor (i.e, Renal Cell Carcinoma (RCC), malignancies other than RCC were excluded) and benign lesion (AML, Oncocytoma, benign cyst). Other pathological features as Adrenal Invasion and Perinephric Fat Extension were documented in less than 2% of the patients and therefore were not included.

Operative variables included the type of operation (open or laparoscopic), renal ischemia (clamping of the renal artery, Yes/No) and ischemia time data. Complications included post-operative urinary leak, delayed bleeding (hematuria or flank hematoma), post-operative renal failure, development of pseudoaneurysm and PBT administration. PBT was defined as transfusion of allogeneic red blood on the day of operation or within the postoperative hospitalization. Post-operative renal failure was defined by any increase in serum creatinine of ≥1.5-fold from the pre-operative baseline.

Statistical analysis was performed using Statistical Package for Social Sciences (SPSS, Version 18.0, Chicago, IL, USA). One-way ANOVA was used for analysis of continuous variables and the chi-square test was used for analysis of categorical variables. A P-value of less than 0.05 was considered statistically significant.

## Result

Six hundred and fifty seven patients underwent partial nephrectomy during the study period. Table 1 lists baseline demographics for the entire study cohort. Of the 657 patients, 87 patients (13.3%) received perioperative blood transfusion. The median number of RBC units was 2. Urinary leak was observed in 17 patients (2.6%).

### Sutures alone vs Sutures and HA

The demographic data of the two groups were comparable in terms of age, gender, Body Mass Index (BMI), kidney side, tumor location along the kidney (i.e, upper or lower pole) and tumor size. Both groups had a similar number of patients with central and hilar tumors, rate of malignant tumors (RCC) and similar proportion of patients undergoing renal artery clamping. The use of a HA did not show a statistically significant difference in warm ischemia time (14.5 vs 19.7 minutes, p =C NS). Furthermore, overall complications did not differ between groups. Specifically, addition of HA did not reduce the rate of PBT (4.1% vs 7.7%, p = NS), delayed bleeding or flank hematomas (2 patients in HAs group and 5 in the parallel group, p = NS). No case of pseudoaneurysm was recorded in any of the patients receiving HAs in comparison to only one case in the parallel group (p = NS). No differences were observed in the rate of post-operative renal failure (Table 2).

### Sutures alone vs Sutures and SURGICEL

Univariate analysis revealed that the two groups were comparable in age, gender, BMI, preoperative HB levels and rate of RCC. However, patients who received SURGICEL had significantly larger tumors in comparison to sutures alone (3.8 cm and 3.08 cm respectively, p = 0.03), higher rate of central renal lesions (43.2% vs 23.8%, p < 0.001) and lower rate of renal vascular clamping (69.5% vs 85.5%, p = 0.004). The majority of patients in the SURGICEL group were operated upon by an open approach (89.6% vs 36.7%, p = 0.001). In regards to complications, analysis of these sub-groups revealed that patients in the SURGICEL group had significantly higher PBT rate (p < 0.001) and higher prevalence of urinary leak cases (p < 0.05). No differences were observed in regards to delayed bleeding (including hematuria or flank hematoma). Notably, a higher incidence rate of pseudoaneurysm events was recorded in the SURGICEL group in comparison to sutures alone (3.3% vs 0.7% respectively, p = NS). No differences were observed in the rate of post-operative renal failure (Table 2).

### Sutures, HA and SURGICEL vs Sutures and SURGICEL

The two groups were comparable in age, gender, BMI, preoperative HB levels and rate of RCC. The group of patients who received HA in addition to SURGICEL had significantly smaller tumors in comparison to the ‘HA free’ patients (3.21 and 3.8 cm respectively, P = 0.001), lower prevalence of central renal lesions (P = 0.03) and higher rate of renal vascular clamping (P = 0.001). The majority of patients in this group were operated upon by a laparoscopic approach. The addition of HA to SURGICEL did not show a statistically significant difference in the rate of PBT (14.6% and 19.1%, P = NS), the prevalence of urinary leak (2.3% and 4.9%, P = NS) or the rate of delayed bleeding including the rate of hematuria, flank hematomas and pseudoaneurysms. Additionally, no differences were observed in the rate of post-operative renal failure (Table 3).

### Analysis based on surgical approach (Open vs. Laparoscopic)

In subgroup analysis of the *laparoscopic* approach alone, the two groups of patients were similar in all clinicopathological variables, including mass size, prevalence of central tumors, tumor location and rate of RCC. The only different variable was rate of renal vascular clamping. The majority of patients who received both SURGICEL and HAs, had significantly higher rate of clamping in comparison to SURGICEL alone (88.6% vs 52.6%, P = 0.001), yet no differences were observed in ischemia time. Comparison of the complication rate did not reveal any advantage to the addition of HA to SURGICEL in regards to PBT rate or post-operative bleeding. However, in the laparoscopic group, the addition of HA to SURGICEL decreased significantly the rate of leaks requiring stent insertion (P = 0.002). Yet, no advantage to HAs in comparison to sutures alone in regards to hemorrhagic complications (including rate of PBT) or the rate of urinary leak was found (Table 4).

Similar results were observed when clinical features and outcomes were compared in patients operated upon by an open approach. Since the number of patients in the ‘Sutures and HA’ group that were operated by an open approach was too small (n = 5), this group was not compared to sutures alone (Table 5). Further analysis of the complication rate only in patients in which renal artery clamping was not performed revealed no advantage in the use of HA, both in comparison to sutures alone or sutures combined with SURGICEL.

### Cryoprecipitate Thrombin vs commercially prepared sealants

This Study included 263 patients treated with Cryoprecipitate thrombin and SURGICEL whereas 38 were treated with both commercial HA and SURGICEL. No differences were found in regards to PBT rate or incidence of urinary leak between the 2 groups. Patients were further divided based on the type of HA used. Eighteen patients were treated with Cryoprecipitate thrombin alone and 8 with commercial sealants. No differences were observed in regards to PBT rate or incidence of urinary leak between the 2 groups.

## Discussion

The use of tissue sealants and glues as HAs while operating or in the operation of PN has gained popularity. However, despite their growing clinical application, their significance in preventing hemorrhage and urinary leak is not evidence based and the decision of using it depends mainly on individual experience. In 2007, Breda *et al*.^13^, reported the results of a large multi-institutional survey, examining the usage patterns of HAs in LPN. The study included 18 centers from Europe and the United States in 1347 LPN cases. Breda and colleagues demonstrated, that up to 80% of urologists used the assistance of one or more HAs intraoperatively. Moreover, of the 18 centers, parenchymal suturing over a bolster of SURGICEL was consistently used by 16 centers. The investigators concluded that although there appears to be some advantage in the use of these agents, they should be limited to control minor bleeding in conjunction with other measurements, including parenchymal suturing over a bolster. However, despite these recommendations, it seems that the use of HAs is still vast, even though there is limited data supporting their effectiveness. In the current study we tried to examine the value of using HAs in PN. Following Breda *et al*., we further analyzed its value with and without the use of SURGICEL. In this study, the use of HAs did not improve or alter the rate of perioperative or post-operative bleeding as well as the rate of urinary leak when compared to sutures alone. When compared to SURGICEL (Table 3) despite the “advantages” in the tumor features (smaller tumors, less centrally located, laparoscopic approach) the addition of HA to the SURGICEL did not reduce the rate of complications, including urinary leak or the need for PBT. Moreover, since vascular clamping was previously associated with decreased hemorrhagic complications, and specifically lower PBT rate^12^, we further analyzed the theoretical advantage of HA to sutures and SURGICEL in patients in which renal artery clamping was not performed. Such analysis revealed no advantage with the use of HA, both in comparison to sutures alone or sutures combined with SURGICEL.

In regards to surgical approach, most studies thus far have examined the beneficial effects of these agents, specifically in LPN. In the current study, we included also patients undergoing OPN. LPN is technically challenging and requires advanced laparoscopic skills. Hence, the laparoscopic approach is often reserved for small, peripheric, exhophitic tumors. In this study, patients operated via an open approach had significantly larger, centrally located tumors and significantly lower rate of vascular clamping (data not shown). Interestingly, even in this “high risk” subgroup of patients, the addition of HAs over a bolster of SURGICEL did not reveal even a slight advantage in any of the examined outcomes.

In addition to the main theoretical benefit of HAs in minimizing postoperative bleeding and decreasing the rate of postoperative blood transfusion, HAs were also suggested to limit warm ischemia time by decreasing the amount of intracorporeal suturing and in some cases potentially promote collecting system healing and reduce postoperative urinary leak^13^. The lack of advantages in any of these aspects in the current cohort, raises the question as to whether HAs should still be used in patients undergoing PN. Supporting these conclusions, a recent study by Cohen *et al*., analyzed the use of a specific type of HAs, Fibrin sealants, during robot-assisted partial nephrectomy (RAPN). Cohen and colleagues demonstrated that the addition of Fibrin glue to hemostatic suture closure does not decrease the rate of complications, blood loss, or hospital stay. Furthermore, no impact was seen on operative time, ischemia time, or other negative outcomes. Consequently, the authors suggested that omitting these agents during RAPN could be safe and cost-effective^14^,^15^. The absolute cost per case may vary between $100–$500, depending on the agent used. At high capacity centers where PNs are routinely performed, the overall cost can be significant. Taking the economic burden into consideration, along with the lack of proven benefits, lead us to the conclusion that the use of HAs, on the whole, is unnecessary in PN patients. We believe that if haemostasis is done well with stitches alone, there is no need for additional adhesive agents. The lack of standardized indications for using HAs vs sutures alone represents some weakness in our study design. However, we believe that this weakness is partly overcome by the fact that all operations were performed in the same surgical environment (four surgeons operating in one medical center) and thus it is reasonable to assume that the decision of using HAs was derived from similar clinical judgment and services routines. Prospective trials would be helpful in interpreting the practicality of these agents.

Limitations of this study include the sample size and the inherent bias associated with its retrospective design. Moreover, the lack of definitive indications for the use of HA’s represent some weakness in our study design. However, we believe that this flaw is partly overcome by the fact that all operations were performed in the same surgical environment, in a high-volume tertiary care institute and thus it is reasonable to assume that the decision on using HA’s was derived from similar clinical judgment and service routines.

## Conclusions

In the current study, there was no difference in complication rates using HAs compared with those without. The use of HAs during open and laparoscopic PN does not impact negative outcomes. A proper suture renorrhaphy during partial nephrectomy may be enough to prevent hemorrhagic complications and urine leak. Further study is necessary to support these findings.

## Additional Information

**How to cite this article**: Abu-Ghanem, Y. *et al*. The use of Haemostatic Agents does not impact the rate of hemorrhagic complications in patients undergoing partial nephrectomy for renal masses. *Sci. Rep*. **6**, 32376; doi: 10.1038/srep32376 (2016).

## Figures and Tables

**Table 1:** Clinicopathologic demographics of 657 patients included in the study.

Variable	No. (%)
Age (years) (median ± sd)	61.85 ± 12.6
Gender	
Male	437 (66.5)
Female	220 (33.5)
BMI	27.1 ± 4.8
Preoperative HB (g/dL)	13.6 ± 1.50
Preoperative Serum Creatinine	1.02 ± 0.37
Central renal lesion	216 (32.9)
Mass location	
Upper Pole	168 (25.6)
Middle aspect	120 (18.3)
Lower Pole	206 (31.4)
Mixed	163 (24.7)
Kidney side	
Left Kidney	329 (50.2)
Right Kidney	326 (49.8)
Tumor size	3.0 ± 1.49
<4 cm	517 (78.7)
>4 cm	140 (21.3)
Pathology	
Malignant (RCC)	493 (75)
Non-malignant*	164 (25)
Operative method	
Open	269 (40.9)
Laparoscopic	388 (59.1)
Renal vascular clamping	519 (78.9)
Ischemia time	20.0 ± 8.6
Renorrhaphy	
Sutures alone	147
Sutures and HA	25
Sutures and SURGICEL	183
Suture, HA and SURGICEL	301
PBT	87 (13.3)
Leak	17 (2.6)
Delayed bleeding	
Hematuria	10 (1.5)
Flank hematoma	6 (0.91)
Pseudoaneurysm	16 (2.4)
Renal Failure	12 (1.8)
DVT/PE	5 (0.76)

Values in parentheses are percentages; Abbreviations: PBT = Perioperative blood transfusion; BMI = Body Mass Index; HB = Hemoglobin; RCC = Renal Cell Carcinoma; HA = Hemostatic agents; DVT = deep vein thrombosis; PE = pulmonary embolism. *Angiomyolipoma (AML), Oncocytoma, simple cyst.

**Table 2:** Univariate analysis - clinical, demographic, operative, and perioperative data of patients undergoing Partial Nephrectomy with only parenchymal suture vs: parenchymal suture with hemostatic agents and SURGICEL, suture with only HA and sutures with only SURGICEL.

Variable	Sutures alone(n = 147)	Sutures and HA(n = 26)	P value	Sutures and SURGICEL(n = 183)	P value
Age (years)	58.7 ± 13.5	64.3 ± 9.7	0.112	61.6 ± 13.5	0.887
Gender					
Male	97 (66)	19 (73.1)	0.761	117 (63.9)	0.698
Female	50 (34)	7 (26.9)		66 (36.1)	
BMI	26.1 ± 5.6	25.9 ± 4.02	0.164	27.7 ± 4.7	0.612
Mass location					
Upper Pole	42 (28.6)	3 (11.5)	0.071	53 (29)	0.135
Middle aspect	22 (15)	5 (19.2)		38 (20.8)	
Lower Pole	38 (25.9)	12 (46.2)		55 (30.1)	
Mixed	45 (30.6)	6 (23.1)		37 (20.2)	
Multifocal mass	16 (10.9)	3 (11.5)	0.922	32 (17.5)	0.091
Kidney side					
Left Kidney	71 (48.3)	16 (61.5)	0.213	87 (47.5)	0.887
Right Kidney	76 (51.7)	10 (38.5)		96 (52.5)	
Central renal lesion	35 (23.8)	9 (34.6)	0.243	79 (43.2)	<0.001
Hilar renal lesion	16 (10.9)	3 (11.5)	0.922	31 (16.9)	0.118
Mass size	3.01 ± 1.43	2.47 ± 1.1	0.727	3.83 ± 1.62	0.003
<4 cm	135 (91.8)	24 (92,3)		117 (63.9)	
>4 cm	12 (8.2)	2 (7.7)		66 (36.1)	
Pathology					
Malignant (RCC)	104 (70.7)	20 (76.9)	0.59	140 (76.5)	0.61
Non-malignant*	43 (29.3)	6 (23.1)		43 (23.5)	
Operative method					
Open	54 (36.7)	5 (19.2)	0.083	164 (89.6)	<0.001
Laparoscopic	93 (63.3)	21 (80.8)		19 (10.4)	
Renal vascular clamping					
Yes	107 (72.8)	21 (80.8)	0.393	127 (69.4)	0.004
No	40 (27.2)	5 (19.2)		56 (30.6)	
Ischemia Time (min)	19.7 ± 10.9	14.5 ± 8.5	0.283	15.8 ± 10.6	0.87
Preoperative HB (g/dL)	13.6 ± 1.5	13.8 ± 1.1	0.09	13.5 ± 1.55	0.899
Discharge HB (g/dL)	11.4 ± 1.5	11.8 ± 1.5	0.845	11.45 ± 1.35	0.261
PBT	6 (4.1)	2 (7.7)	0.419	35 (19.1)	<0.001
Leak	1 (0.7)	0 (0)	0.673	9 (4.9)	0.026
Delayed bleeding					
Hematuria	3 (2.04)	1 (3.8)	0.576	2 (1.6)	0.867
Flank hematoma	0 (0)	0 (0)	0.773	1 (0.5)	NA
Pseudoaneurysm	1 (0.7)	0 (0)	NA	5 (2.7)	0.836
Renal Failure	3 (2.04)	0 (0)	NA	6 (3.3)	0.718
DVT/PE	0 (0)	1 (3.8)	NA	3 (0.55)	NA

Values in parentheses are percentages; Abbreviations: PBT = Perioperative blood transfusion; BMI = Body Mass Index; HB = Hemoglobin; RCC = Renal Cell Carcinoma; HA = Hemostatic agents; DVT = deep vein thrombosis; PE = pulmonary embolism. *Angiomyolipoma (AML), Oncocytoma, simple cyst.

**Table 3:** Univariate analysis - clinical, demographic, operative, and perioperative data of patients undergoing Partial Nephrectomy with only parenchymal suture, or parenchymal suture with SURGICEL and Hemostatic agens.

Variable	Suture, HA and SURGICEL(n = 301)	Sutures and SURGICEL(n = 183)	P value
Age (years)	61.5 ± 11.6	61.6 ± 13.5	0.079
Gender			
Male	204 (67.8)	117 (63.9)	0.386
Female	97 (32.2)	66 (36.1)	
BMI	28.1 ± 4.8	27.7 ± 4.7	0.853
Mass location			0.368
Upper Pole	53 (29)	70 (23.2)	
Middle aspect	38 (20.8)	55 (18.3)	
Lower Pole	55 (30)	101 (33.5)	
Mixed	37 (20.2)	75 (25)	
Multifocal mass	14 (4.7)	32 (17.5)	0.123
Kidney side			0.359
Left Kidney	156 (51.8)	87 (47.5)	
Right Kidney	145 (48.2)	96 (52.5)	
Central renal lesion	101 (33.6)	79 (43.2)	0.034
Hilar renal lesion	40 (13.3)	31 (16.9)	0.271
Mass size	3.21 ± 1.4	3.8 ± 1.6	<0.001
<4cm	241 (80.1)	117 (63.9)	
>4cm	60 (19.9)	66 (36.1)	
Pathology			
Malignant (RCC)	229 (76.1)	140 (76.5)	0.92
Non-malignant*	72 (23.9)	43 (23.5)	
Operative method			<0.001
Open	46 (15.3)	164 (89.6)	
Laparoscopic	255 (84.7)	19 (10.4)	
Renal vascular clamping			<0.001
Yes	269 (89.4)	127 (69.4)	
No	32 (10.6)	56 (30.6)	
Ischemia Time (min)	22.5 ± 10.3	14.8 ± 10.6	0.950
Preoperative HB (g/dL)	13.6 ± 1.5	13.5 ± 1.55	0.722
Postoperative HB (g/dL)	11.86 ± 1.5	11.45 ± 1.35	0.473
PBT	44 (14.6)	35 (19.1)	0.193
Leak	7 (2.3)	9 (4.9)	0.122
Delayed bleeding			
Hematuria	4 (1.3) 4	2 (1.6)	0.794
Flank hematoma	(1.3)	1 (0.5)	0.832
Pseudoaneurysm	10 (3.3)	5 (2.7)	0.798
Renal Failure	3 (1.0)	6 (3.3)	0.153
DVT/PE	2 (0.67)	3 (0.55)	0.462

Values in parentheses are percentages; Abbreviations: PBT = Perioperative blood transfusion; BMI = Body Mass Index; HB = Hemoglobin; RCC = Renal Cell Carcinoma; HA = Hemostatic agents; DVT = deep vein thrombosis; PE = pulmonary embolism. *Angiomyolipoma (AML), Oncocytoma, simple cyst.

**Table 4:** Transfusion rates among different variables in patients undergoing laparoscopic PN.

Variable	Suture, HA and SURGICEL (n=255)	Sutures and SURGICEL (n **= **19)	P value	Suture and HA (n **= **21)	Sutures alone (n **= **93)	P value
Age (years)	62 ± 11.3	61.9 ± 15.3	0.347	63.1 ± 9.8	57.6 ± 13.4	0.229
Central renal lesion	74 (29)	2 (10.5)	0.083	5 (23.8)	10 (10.8)	0.110
Mass size	3.04 ± 1.3	3.03 ± 1.6	0.127	2.43 ± 1.2	2.7 ± 0.96 89	0.333
<4cm	218 (85.5)	15 (78.9)		19 (90.5)	(95.7)	
>4cm	37 (14.5)	4 (21.1)		2 (9.5)	4 (4.3)	
Pathology						
Malignant (RCC)	198 (77.6)	12 (63.2)	0.15	15 (71.4)	66 (71.0)	0.97
Non-malignant*	57 (22.4)	7 (36.8)		6 (28.6)	27 (29.0)	
Renal vascular clamping			<0.001			0.095
Yes	226 (88.6)	10 (52.6)		19 (90.5)	68 (73.1)	
No	29 (11.4)	9 (47.4)		2 (9.5)	25 (26.9)	
Ischemia Time (min)	25.6 ± 7.8	21.1 ± 13.6	0.496	17.5 ± 6.5	24.8 ± 9.3	0.123
PBT	31 (12.2)	3 (15.8)	0.643	1 (4.8)	3 (3.2)	0.730
Leak	6 (2.4)	3 (15.8)	0.002	0	0	NA
Delayed bleeding						
Hematuria	7	0	NA	0	2	NA
Flank hematoma	6	0	NA	1	0	NA
Pseudoaneurysm	2	0	NA	0	0	NA
Renal Failure	3	0	NA	0	1	NA
DVT/PE	2	0	NA	0	0	NA

Values in parentheses are percentages; Abbreviations: PBT = Perioperative blood transfusion; RCC = Renal Cell Carcinoma; HA = Hemostatic agents; DVT = deep vein thrombosis; PE = pulmonary embolism. *Angiomyolipoma (AML), Oncocytoma, simple cyst.

**Table 5:** Transfusion rates among different variables in patients undergoing open PN.

Variable	Suture, HA and SURGICEL (n **= **46)	Sutures and SURGICEL (n **= **164)	P value
Age (years)	58.9 ± 13	61.6 ± 13.3	0.735
Central renal lesion	27 (58.7)	77 (47)	0.159
Mass size	4.1 ± 1.59	3.9 ± 1.6	0.799
<4cm	23(50)	102 (62.2)	
>4cm	23 (50)	62 (37.8)	
Pathology			
Malignant (RCC)	32 (69.6)	128 (78)	0.16
Non-malignant*	14 (30.4)	36 (22)	
Renal vascular clamping			0.005
Yes	42 (91.3)	47 (28.7)	
No	4 (8.7)	117 (71.3)	
Ischemia Time (min)	18.8 ± 7.5	18.2 ± 7.96	0.813
PBT	13 (28.3)	33 (20.1)	0.238
Leak	1 (2.2)	6 (3.7)	0.620
Delayed bleeding			
Hematuria	1 (2.17)	5 (3.05)	
Flank hematoma	0	2 (1.22)	NA
Pseudoaneurysm	1 (2.17)	2 (1.22)	
Renal Failure	0	6 (3.66)	NA
DVT/PE	0	3 (1.83)	NA

Values in parentheses are percentages; Abbreviations: PBT = Perioperative blood transfusion; RCC = Renal Cell Carcinoma; HA = Hemostatic agents; DVT = deep vein thrombosis; PE = pulmonary embolism. *Angiomyolipoma (AML), Oncocytoma, simple cyst.
